# Evaluation of dry eye findings in patients with vitiligo

**DOI:** 10.12669/pjms.313.6926

**Published:** 2015

**Authors:** Aysun Sanal Dogan, Damla Atacan, Selda Pelin Kartal Durmazlar, Mutlu Acar, Canan Gurdal

**Affiliations:** 1 Aysun Sanal Dogan, Departments of Ophthalmology, Diskapi Yidirim Beyazit Training and Research Hospital, Ministry of Health, Ankara, Turkey; 2 Damla Atacan, Departments of Dermatology, Diskapi Yidirim Beyazit Training and Research Hospital, Ministry of Health, Ankara, Turkey; 3 Selda Pelin Kartal Durmazlar, Departments of Dermatology, Diskapi Yidirim Beyazit Training and Research Hospital, Ministry of Health, Ankara, Turkey; 4 Mutlu Acar, Departments of Ophthalmology, Diskapi Yidirim Beyazit Training and Research Hospital, Ministry of Health, Ankara, Turkey; 5 Canan Gurdal, Departments of Ophthalmology, Diskapi Yidirim Beyazit Training and Research Hospital, Ministry of Health, Ankara, Turkey

**Keywords:** Dry eye, OSDI, Vitiligo

## Abstract

**Objective:**

To evaluate the association of dry eye and vitiligo diseases with objective parameters and a questionnaire.

**Methods:**

The study was conducted in 30 vitiligo patients and 31 patients with non-complicated refractory complaints. All the patients underwent complete ophthalmologic examinations including fluorescein break-up time (FBU), corneal fluorescein staining (CFS) and Schirmer test-I. The dry eye status was evaluated by means of Ocular Surface Disease Index (OSDI).

**Results:**

The groups were similar regarding the age and gender distribution. The vitiligo group had higher OSDI scores (26.1±15.9 vs 14.7±5.4, t-test, p<0.001), shorter FBU (7.8±2.9 vs 9.8±2.2, Mann Whitney U test, p=0.005) and higher CFS positivity (18/30 vs 3/31, chi-square test, p<0.001) than control group. The groups were similar regarding the Meibomian Gland Dysfunction (MGD) and Schirmer test results. Fourteen (46.7%) of vitiligo patients had periocular involvement. The analysis within vitiligo patients revealed that FBU and Schirmer test were shorter in patients with periocular involvement, the OSDI scores and MGD status were similar.

**Conclusion:**

Our study suggest a possible association of dry eye and vitiligo diseases. The diagnostic tools for dry eye disease are in good correlation with each other. The OSDI questionnaire seems practical for both diagnostic purposes and follow-up.

## INTRODUCTION

Dry eye disease, is one of the most common topics of ophthalmologic daily practice. It is most of the time an isolated condition however may present as a part of systemic disorders. This chronic, multifactorial and inflammatory condition develops as a result of altered tear metabolism because of the lacrimal functional unit dysfunction.^1^ Vitiligo is a disease characterized with the appearance of achromic or hypochromic patches on the skin and mucous membranes as well.^2^ Association of vitiligo with several ocular diseases has been described such as uveitis, glaucoma.^3^,^4^ The association of vitiligo with dry eye has not been studied in details. Only one study^5^ investigated this association and reported alterations in ocular surface and tear functions.

Our study aimed to investigate the presence of dry eye in vitiligo patients with objective parameters and Ocular Surface Disease Index (OSDI) questionnaire and compare the results with control group.

## METHODS

The present study was conducted between March 2014 and July 2014 after obtaining the approval of the local ethic committee. Written consent was taken from all of the subjects. Study group consisted of 30 vitiligo patients and the control group included 31 patients. All subjects had come to ophthalmology department with non-complicated refractory complaints. Patients under active vitiligo treatment, with any systemic and ocular diseases and who were on oral or parenteral medication were excluded. All the patients underwent complete ophthalmologic examinations including fluorescein break-up time (FBU), corneal fluorescein staining (CFS) and Schirmer test-l with topical anesthesia. The dry eye status was evaluated by means of OSDI. The CFS staining was graded according to the Oxford scheme and severity of meibomian gland dysfunction (MGD) was classified according to the Bron et al.^6^ system. All procedures were performed by one of the authors (A.S.D.). Though, with practical purposes, these two parameters were analyzed as dichotomous variables (present or absent) because of the small number of patients. The data were recorded in a standard sheet and statistical analysis was done by SPSS 15.0 program.

## RESULTS

Study (Group V) and control (Group C) groups were similar regarding the age (43.3±12.1 vs 39.6±11.8 years, t-test, p=0.230) and gender distribution (male/female= 12/18 vs 12/19, chi-square test, p=0.918). The OSDI scores were higher, FBU was shorter and CFS positivity was higher in vitiligo patients where groups were similar regarding the MGD status and Schirmer test results (Table-I). The periocular involvement was present in 14 (46.7%) of vitiligo patients. When we analyzed the vitiligo patients regarding the periocular involvement state, we detected that patients with periocular involvement, although statistically insignificant, were younger than the patients without periocular involvement (39.6±12.9 years vs 46.5±10.7 years, t-test, p= 0.120).

**Table-I:** Comparison of results of two groups (61 patients).

	Vitiligo (n= 30)		Control (n=31)		P value	
	Right eye	Left eye	Right eye	Left eye	Right eye	Left eye
FBU (seconds)	7.8±2.9	8.2±2.9	9.8±2.2	10±2.2	0.005^a^	0.003^a^
CFS * (0/1/2/3)	12/10/6/2	15/8/6/1	28/3/0/0	26/5/0/0	<0.001^b^	<0.005^b^
MGD * (1/2/3/4/5)	5/16/6/3/0	6/15/6/3/0	6/22/3/0/0	7/20/3/1/0	0.785^b^	0.806^b^
Schirmer test (mm)	9.8±2.6	10±2.9	11.4±3.2	11.6±3.1	0.055^a^	0.089^a^
OSDI score	26.1±15.9		14.7±5.4		<0.001^c^	

a: Mann-Whitney U test,

b: Chi-square test,

c: t-test.

*: comparison was done regarding absence versus presence status.

In vitiligo patients, FBU was shorter than 10 seconds in 70% (right eye) and 70% (left eye) when compared with normals (48.4% for right eye, chi-square test, p=0.086 and 38.7% for left eye, chi-square test, p= 0.014). The analysis of the study parameters within vitiligo patients revealed that FBU and Schirmer test were shorter in patients with periocular involvement, the OSDI scores and MGD status were similar, the presence of CFS was more prominent although being statistically insignificant (Table-II).

**Table-II:** Comparison of results within vitiligo patients regarding the periocular (VPO+/-) involvement (30 patients).

	Group VPO(+) (n= 14)		Group VPO(-) (n=16)		P value	
	Right eye	Left eye	Right eye	Left eye	Right eye	Left eye
FBU (seconds)	6.6±2.7	7.1±2.4	8.9±2.6	9.2±2.9	0.034^a^	0.052^a^
CFS * status (absent/present)	3/11	4/10	9/7	11/5	0.072^b^	0.066^b^
MGD* (absent/present)	2/12	3/11	3/13	3/13	1.000^b^	1.000^b^
Schirmer test (mm)	8.4±2.8	8.9±3.1	10.9±1.8	11.0±2.3	0.007^a^	0.043^a^
OSDI score	29.3±16.7		23.3±15.2		0.310^c^	

a: Mann-Whitney U test,

b: Chi-square test,

c: t-test.

*: comparison was done regarding absence versus presence status.

In our study, we detected that OSDI scores, FBU, CFS and Schirmer test were all correlated with each other excellently (Table-III).

**Table-III:** The correlations between OSDI scores and other objective tests (61 patients).

	Correlation coefficient (Spearman’s rho)	Significance (p value)
OSDI scores - FBU right eye	-0.508	<0.001
OSDI scores - FBU left eye	-0.503	<0.001
OSDI scores - CFS right eye	+0.632	<0.001
OSDI scores - CFS left eye	+0.629	<0.001
OSDI scores - Schirmer right eye	-0.349	0.006
OSDI scores - Schirmer left eye	-0.452	0.002

## DISCUSSION

Vitiligo is one of the important diseases of dermatology and has a prevalence of up to 2% in the population.^7^ There is no specific national epidemiological study on the prevalence of vitiligo. However, national studies those depending on hospital registries reported prevalences of 1.4% in pediatric^8^ and 2.1% in adult populations.^9^ It is a chronic disease which requires a regular follow-up and treatment. The dry eye syndrome is another important topic of the ophthalmologic practice which has a prevalence rate up to 15%.^10^ The information about the national prevalence of dry eye disease is lacking. The data obtained from the field scanning phase by questionnaires of one national specific epidemiological study on primary Sjögren’s Syndrome revealed that 35.2% of subjects reported persistent dry eye complaints for more than 3 months and 9.4% reported routine use of tear substitute eye drops.^11^ Both diseases have an autoimmune inflammatory basis and therefore it will not be unwise to expect a significant co-incidence. The ophthalmologic consequences have been studied widely and uveitis has become most studied subject because of its autoimmune inflammatory pathophysiology.^12^ However, a more prevalent disease, dry eye syndrome’s association with vitiligo has not been studied as much as it deserved, probably because of its benign course and underestimation by both practitioners and the patients.

The present study was conducted in order to investigate this association, to increase the awareness about the dry eye disease which disturbs the personal quality of life and to assess the performance of OSDI tool in this particular patient group. Our results in this patient population without manifested dry eye complaints, the objective and subjective tests showed that vitiligo patients had significant difference from the control group which should attract the attention of both practitioners and patients.

The diagnosis of dry eye is mostly dependent on the patients’ complaints and confirmation may be done by objective parameters such as FBU, CFS and Schirmer tests. These tests have variable degrees of sensitivities and specificities.^1^ Questionnaires are useful in daily practice that eases the diagnosis and the assessment of response to treatment. Our findings revealed that FBU was shorter, CFS was present more frequently and Schirmer test (although did not reach statistical significance) was shorter in vitiligo patients which made us think that there was a tendency for dry eye syndrome in these patients. Supporting these findings, the OSDI score was higher in vitiligo patients. There are several cut-off points for these diagnostic tools. Since, the results of diagnostic tests of dry eye disease present a broad range of variability among different conditions, depending on one cut-off point is not reliable.^13^ In a recent study, Alves *et al*. reported that vital staining and FBU correlated best with one another whereas the best test combination to detect dry eye disease was: OSDI/FBU/Schirmer.^13^

Our results also supported that the correlation was excellent between the mentioned tests. We abstained to categorize our results with these limiting cut-off points since none of our patient and control groups had manifested dry eye complaints. Therefore, we preferred to make a quantitative comparison instead of a qualitative way. When we interpret our result with this point of view, we can state that there is a tendency for dry eye disease in vitiligo patients as compared with the normal population. For instance, the OSDI scores, although being under the cut-off point (35 points), there is a significant increase in vitiligo patients (26.1 vs 14.7). Another parameter is the FBU which has results around the predescribed cut-off (10 seconds) point (Table-I). However, the time was shorter in vitiligo patients.

The facial involvement may be present up to 87% of generalized vitiligo patients with various severity.^14^ The periocular involvement is reported to be present in 60% of cases. In our study group of vitiligo patients which all have generalized form, 47% of patients had periocular involvement. It’s shown that at perilesional zones of vitiligo areas there is apoptosis of melonocytes, and accumulation of T lymphocytes.^15^ It seems to be logical to predict more ocular surface consequences of vitiligo in patients with periocular involvement. Therefore, we made more detailed analysis in patients with periocular involvement. Our analysis in the vitiligo group revealed that FBU and Schirmer test was shorter and CFS was more prominent (p=0.072 and p=0.066) in patients with periocular involvement though the OSDI scores were similar. These findings support the localization of the lesion might have an impact on the pathophysiology. There are not many studies investigating the dry eye status in details in vitiligo patients in national or global level. There is one unique study which is from Turkey on the association of dry eye and vitiligo disease that reported similar findings in terms of FBU results.^5^ The authors used FBU and Schirmer tests, however the complaints were not evaluated by means of OSDI.^5^ Our findings with the present literature data make us think to hypothesize a significant relation between dry eye and vitiligo diseases. Speeckaert *et al*, reported that patients with periocular involvement were younger in their retrospective cohort study in 700 patients.^14^ In our series, similarly, periocular involved patients were younger (39.6±12.9 years vs 46.5±10.7 years). However, this difference was not statistically significant which might be attributed to the small number of patients.

The most studied ocular disease in vitiligo patients was uveitis since the last 30 years because of the probable common autoimmune pathophysiology.^12^ Our study has very few things to say on this issue because of the study design. We excluded the patients with known autoimmune or any other systemic diseases other than vitiligo. Moreover, another exclusion criteria was the active vitiligo treatment within the last one year. These exclusion criterias were put in order to prevent the data contamination. Dry eye disease is basically categorized in two types: 1. tear deficiency, 2. evaporative.^1^ MGD is the leading cause of evaporative type dry eye disease.^16^

In our series, groups were similar regarding the MGD status. Therefore, absence of MGD effect in our study group supports the lacrimal unit dysfunction as a causative factor which causes alteration in tear metabolism. There are several possible etiological factors underlying this dysfunction such as age, hormonal, environmental factors, systemic or local autoimmune diseases, topical medications, contact lens wear and surgery which may all cause an inflammatory response.^17^,^18^ However, as far as we eliminated the other possible factors, our findings which resulted from the comparison of vitiligo and normal groups made us to hypothesize a common autoimmune or inflammatory etiology where the analysis within vitiligo patients directed us to hypothesize the localization of the lesion may have an impact on the development of dry eye that a local inflammatory theory might be suggested. Therefore, more detailed studies at the level of inflammatory products in different patient groups should be conducted. Our study provides common and acceptable hints for these kinds of future studies.

We think that one of the most important outcomes of our study is the utilization of OSDI tool for dry eye disease in a different patient group. To the best our knowledge, our study is the first in the literature which utilized the OSDI in vitiligo patients. Our series revealed that OSDI has a good correlation with other diagnostic tools. These results on OSDI scores showed that dry eye may present as a part of a dermatological disease and it can be used by the practitioners of different disciplines as a simple and quick scanning way. Patients can be referred to ophthalmologists and required suggestions may be recommended before the disturbing complaints become manifested.

## CONCLUSION

Our study suggests association of dry eye and vitiligo diseases. The diagnostic tools for dry eye disease are in good correlation with each other which also has been proven in a different patient group. The OSDI questionnaire seems practical for both diagnostic and follow-up purposes which can also be used by other disciplines of medicine.
